# Nano-La_2_O_3_ Induces Honeybee (*Apis mellifera*) Death and Enriches for Pathogens in Honeybee Gut Bacterial Communities

**DOI:** 10.3389/fmicb.2021.780943

**Published:** 2021-12-02

**Authors:** Yong-Jun Liu, Zhongwang Jing, Xue-Ting Bai, Qing-Yun Diao, Jichen Wang, Yan-Yan Wu, Qing Zhao, Tian Xia, Baoshan Xing, Patricia A. Holden, Yuan Ge

**Affiliations:** ^1^Key Laboratory of Pollinating Insect Biology, Institute of Apicultural Research, Chinese Academy of Agricultural Sciences, Beijing, China; ^2^State Key Laboratory of Urban and Regional Ecology, Research Center for Eco-Environmental Sciences, Chinese Academy of Sciences, Beijing, China; ^3^University of Chinese Academy of Sciences, Beijing, China; ^4^Key Laboratory of Pollution Ecology and Environmental Engineering, Institute of Applied Ecology, Chinese Academy of Sciences, Shenyang, China; ^5^Division of NanoMedicine, Department of Medicine, University of California, Los Angeles, Los Angeles, CA, United States; ^6^Stockbridge School of Agriculture, University of Massachusetts, Amherst, MA, United States; ^7^Bren School of Environmental Science & Management, Earth Research Institute, University of California, Santa Barbara, Santa Barbara, CA, United States

**Keywords:** *Apis mellifera*, rare earth oxide nanoparticles, honeybee gut microbiota, nano-La_2_O_3_, honeybee health

## Abstract

Honeybees (*Apis mellifera*) can be exposed *via* numerous potential pathways to ambient nanoparticles (NPs), including rare earth oxide (REO) NPs that are increasingly used and released into the environment. Gut microorganisms are pivotal in mediating honeybee health, but how REO NPs may affect honeybee health and gut microbiota remains poorly understood. To address this knowledge gap, honeybees were fed pollen and sucrose syrup containing 0, 1, 10, 100, and 1000mgkg^−1^ of nano-La_2_O_3_ for 12days. Nano-La_2_O_3_ exerted detrimental effects on honeybee physiology, as reflected by dose-dependent adverse effects of nano-La_2_O_3_ on survival, pollen consumption, and body weight (*p*<0.05). Nano-La_2_O_3_ caused the dysbiosis of honeybee gut bacterial communities, as evidenced by the change of gut bacterial community composition, the enrichment of pathogenic *Serratia* and *Frischella*, and the alteration of digestion-related taxa *Bombella* (*p*<0.05). There were significant correlations between honeybee physiological parameters and the relative abundances of pathogenic *Serratia* and *Frischella* (*p*<0.05), underscoring linkages between honeybee health and gut bacterial communities. Taken together, this study demonstrates that nano-La_2_O_3_ can cause detrimental effects on honeybee health, potentially by disordering gut bacterial communities. This study thus reveals a previously overlooked effect of nano-La_2_O_3_ on the ecologically and economically important honeybee species *Apis mellifera*.

## Introduction

Honeybees (e.g., *Apis mellifera*) provide essential pollination services for agricultural ecosystems and valuable apiary products for human nutritional needs (Klein et al., 2007). Due to their extensive social activities within 14km^2^ foraging areas, honeybees are exposed to conventional contaminants, for example, pesticides, antibiotics, and respirable suspended particulate matters, that may lead to the decline of honeybee colonies or the deterioration of honeybee health status (Bargańska et al., 2016; Thimmegowda et al., 2020; Bondarenko et al., 2021; Kapoor et al., 2021). Among the emerging contaminants, rare earth oxide (REO) nanoparticles (NPs), characterized by their unique chemical and physical properties, have been one of most widely used materials in various industries and biotechnology applications (Mastronardi et al., 2015; Gao et al., 2017; Kos et al., 2017). For example, the fertilizers, pesticides, and germination stimulants containing or engineered with REO NPs have been widely used to enhance the efficiency and sustainability of agricultural practices, and nano-La_2_O_3_ is reported to account for approximately 30% of REO NPs additives and have higher cytotoxicity compared with other REO NPs (De la Torre Roche et al., 2015; Mastronardi et al., 2015; Servin et al., 2015; Gao et al., 2017). This make honeybees highly susceptible to the exposure and toxicity of REO NPs, through contacting with and ingesting these particles directly or indirectly from the surrounding environments, especially the agricultural ones, such as plant and flower surfaces, pollen and nectar, and soil and dust (Ma et al., 2011; De la Torre Roche et al., 2015; Kos et al., 2017; Radziwill-Bienkowska et al., 2018). Once soil was contaminated by nano-La_2_O_3_, plants can serve as a potential intermediary pathway that could bioaccumulate and transport them to primary consumers, for example, *Acheta domestic*a, *Tenebrionoidea*, and honeybees (Ma et al., 2011; De la Torre Roche et al., 2015). Related studies have also demonstrated that honeybees come in contact with metal oxide NPs (e.g., CeO_2_, CdO, and PbO) through surface particle adhesion, dust inhalation, foraging on contaminated food, or water (Kos et al., 2017; Al Naggar et al., 2020). Therefore, honeybees may suffer the environmental exposure of terrestrial REO NPs and serve as sensitive indicators of environmental quality. Despite the fact that honeybee gut microorganisms take important roles in maintaining host immunity, metabolism, and health (Kwong and Moran, 2016), it is unknown whether and how REO NPs exposure will cause deterioration of honeybee health and dysbiosis of honeybee gut microbiota and whether the gut dysbiosis will further mediate the toxic effect of environmental contaminants on honeybees health.

Although there are few studies regarding effects of REO NPs on honeybee health, related research implies that metal oxide NPs may adversely affect honeybee health, through mechanisms relating to, for example, signals blocking, reactive oxygen species (ROS), and cell damage (Nel et al., 2006; Benelli, 2018). For example, the mortality rate of *Apis mellifera* increased with exposure concentrations of REO NPs (nano-TiO_2_, nano-ZnO-TiO_2_, and nano-Ag-TiO_2_), implying a dose-dependent toxic effect of metal oxide NPs on honeybee (Dabour et al., 2019). Also, exposure to nano-CeO_2_ causes undesirable neurological effects on honeybee *Apis mellifera*, by inhibiting the activity of membrane acetylcholinesterase (AChE) which further influences cholinergic function of the nervous system (Kos et al., 2017). Also, nano-CdO and nano-PbO can enhance ROS production and thus cause free radical-induced oxidative damage to honeybee *Apis millefera*, accompanied by anti-oxidative responses, for example, increased catalase production (Al Naggar et al., 2020).

REO NPs may also disturb honeybee gut microbiota. Previous studies have shown the undesirable effects of various types of NPs on the soil microbiomes and the gut microbiota of animals and insects (Han et al., 2014; Ge et al., 2016; Zhu et al., 2018; Chen et al., 2021). For instance, nano-ZnO and carbonaceous nanoparticles disturb the soil bacterial community structure and change functionally important microbial groups associated with C, N, and S cycling (Ge et al., 2018; Xu et al., 2018a; Wu et al., 2019). Silver NPs alter the gut bacterial communities of *Drosophila* and *Collembola* (Han et al., 2014; Zhu et al., 2018). But, it is still unclear whether, and how, REO NPs affect gut microbiota of honeybees, specifically the model species *Apis mellifera*. Honeybee gut microbiota could be influenced by various factors, including pathogens, antibiotics, pesticides, diet, and host attributes and environmental conditions (Cox-Foster et al., 2007; Raymann et al., 2017; Liu et al., 2019; Ge et al., 2021). Previous study has demonstrated that the shifts of gut bacterial communities in bumblebees may serve as a characteristic of disease states, featuring as low abundance of core species and high incidence of opportunistic environmental bacteria (Cariveau et al., 2014). Given the significant roles of honeybee gut microbiota in maintaining host health and fitness (Kwong and Moran, 2016; Zhang et al., 2020) and the likelihood of honeybee exposure to REOs *via* ingestion, it is imperative to understand the ecological effects of REO NPs on honeybee gut microbiota to guide the safe design and application of REO NPs.

In this study, the aims were to (1) investigate the toxicity of REO NPs on honeybee health and gut bacterial communities and (2) explore the relationship between REO NP exposure, gut bacterial communities, and host responses. The working hypotheses were that REO NPs would, in a dose-dependent fashion, disturb honeybee gut microbiota and also directly impact honeybee physiology. A further hypothesis was that the overall effects of REO NPs across physiology and gut microbial effects would be additive. To test these hypotheses, honeybees were fed food amended with different concentrations of nano-La_2_O_3_. Here, nano-La_2_O_3_ was used as a representative REO NP because of its multifunctionality and high cytotoxicity (De la Torre Roche et al., 2015; Gao et al., 2017). Honeybee survival, pollen consumption, and body weight were quantified, and the composition of the honeybee gut bacterial community was investigated. This is the first study to analyze the effects of nano-La_2_O_3_ on honeybees and their gut microbiota, and the results contribute new knowledge regarding the environmental risks of REO NPs.

## Materials and Methods

### The Model Honeybee

Honeybees (*Apis mellifera*) used for nano-La_2_O_3_ exposure experiment were incubated at the Institute of Apicultural Research apiary, Chinese Academy of Agricultural Science^1^ following standard protocols (Liu et al., 2019). Briefly, brood frames of a single hive with capped honeybee pupae were placed in an RXZ-380C climate-controlled incubator (Ningbo, China; 34±1°C, 60±10% relative humidity, in darkness) for up to 12h to obtain honeybee specimens. The newly emerged honeybees (less than 12h old) were randomly divided into five rearing cages with 120 honeybees per cage and further incubated for 1week (30±1°C, 45±5% relative humidity, in darkness) by feeding fresh pollen, sterile sucrose solution (50% wt/wt), and deionized water *ad libitum* to initiate microbial colonization in the gut (Ellegaard and Engel, 2019). After one-week pre-incubation, the adult honeybees were exposed to nano-La_2_O_3_ NPs (Day 0).

### La_2_O_3_ NPs

Nano-La_2_O_3_ (25±5nm) was obtained from the University of California Center for the Environmental Implications of Nanotechnology and characterized in a previous study (Qi et al., 2019). Briefly, the nano-La_2_O_3_ studied was of >99.99% purity and composed of spherical particles that aggregated in deionized water (pH=6.8) to 589±16nm. The zeta potential of this nano-La_2_O_3_ was previously determined to be 9±1mV in deionized water (pH=6.8), and the dissolution extent was determined to be 14% after incubating (24h at 37°C) in an acidic aqueous solution (HCl, 50μgmL^−1^, pH=4.5; Li et al., 2014).

### Experimental Design

Fresh pollen grains were collected from *Camellia sinensis*, freeze-dried under vacuum in a lyophilizer (Songyuan Huaxing, Beijing, China), and ground into a fine powder using a mortar and pestle. To obtain a homogeneous NP distribution, nano-La_2_O_3_ powder was thoroughly mixed with the ground pollen with a handheld kitchen mixer for 10min, diluted to a series of concentrations (2.5, 25, 250, and 2500mgkg^−1^ pollen) using a 10-fold dilution method (Ge et al., 2018), and then stored separately at −20°C for daily use. Before daily dietary exposure, the mixture of pollen and nano-La_2_O_3_ was dispersed (1:1.5 weight ratio) into an aqueous sucrose solution (50% wt/wt in sterile water) to promote ingestion by honeybees (Jack et al., 2016). Therefore, the final target exposure doses of nano-La_2_O_3_ in the mixed pollen and sucrose syrup were 1, 10, 100, and 1,000mgkg^−1^. Negative exposure control was also conducted by treating honeybees with the mixture of pollen (without nano-La_2_O_3_) and sucrose solution (1:1.5 weight ratio).

The exposure doses were chosen to represent several scenarios of dietary exposure: possible environmental concentrations (low or medium concentrations), predicted REO NP environmental hotspots (high concentrations), and potential scenarios (the highest concentrations) based on previous studies (Wen et al., 2001; Tyler, 2004; Gottschalk et al., 2009) and some assumptions. Previous studies reported the concentrations of La in plants (0.004–40mgkg^−1^; Wen et al., 2001; Tyler, 2004), surface soils (5.5–44mgkg^−1^; Tyler, 2004), and sediments (5–321mgkg^−1^; Tyler, 2004; Xu et al., 2018b); and the proportion of oxidation state of La was 35–70% (Wen et al., 2001). Also, La compounds tend to be colloid or nanoclusters (< 200nm) in environmental matrices (Ma et al., 2011; Kulaksız and Bau, 2013), and approximate 1–30% nanoparticles can be isolated from bulk soil (Theng and Yuan, 2008). We thus assumed that 1–30% of the La_2_O_3_ in environmental matrices was nano-La_2_O_3._ Based on this assumption, the estimated concentrations of nano-La_2_O_3_ were calculated as 0.00002–10mgkg^−1^ in plants, 0.02–11mgkg^−1^ in surface soils, and 0.02–79mgkg^−1^ in sediments. Therefore, the low and medium concentrations of nano-La_2_O_3_ (1 and 10mgkg^−1^) used in this study were comparable to the estimated concentrations of nano-La_2_O_3_ in environmental matrices. Considering that NP distributions in terrestrial environments may be highly heterogeneous such that very high concentrations may occur in localized areas (Gottschalk et al., 2009). The highest dose was also regarded as simulating an extreme endmember concentration within ranges of reported simulated or measured metal oxide NP concentrations in terrestrial environments (Holden et al., 2014). Using the highest concentration here of 1,000mgkg^−1^ also allows for examining future potential scenarios of NP environmental buildup, as a situation being previously considered (Priester et al., 2013; De la Torre Roche et al., 2015).

To examine the impacts of nano-La_2_O_3_ ingestion, adult honeybees in five rearing cages in climate-controlled incubator (Ningbo Jiangnan, Ningbo, China; 30±1°C with 45±5% relative humidity, in darkness) were orally exposed to different concentrations of nano-La_2_O_3_ (0, 1, 10, 100, and 1,000mgkg^−1^) for up to 12days (Liu et al., 2019). Each cage contained three sterile Petri dish feeders: one feeder containing 4g of a freshly prepared pollen and sterile sucrose syrup mixture containing 0, 1, 10, 100, and 1,000mgkg^−1^ nano-La_2_O_3_ for dietary exposure; another feeder containing 20g sucrose solution (50% wt/wt in sterile water) to minimize the indirect effect of insufficient feeding; and the third feeder containing 30ml deionized water (Di Pasquale et al., 2013). The amounts provided were more than sufficient for dietary and water needs and were replaced daily with equal amounts during the exposure experiment. A control cage that contained dietary provisions and water, but no honeybees, was also conducted in the incubator simultaneously to measure water evaporation.

### La Residue in Honeybees

To assess the naturally environmental exposure of honeybees to La, 3 wild honeybees were randomly collected using a sweep net in the field within Beijing Botanical Garden where the Institute of Apicultural Research is located. To measure the La residue in honeybees under controlled laboratory conditions, 3 honeybees of each treatment were sacrificed after 12days exposure, with each honeybee serving as a biological replicate. Each honeybee was washed and frozen in liquid nitrogen. For each honeybee, the whole body was added with 5ml concentrated nitric acid (HNO_3_) and 1ml hydrogen peroxide (H_2_O_2_, 30% vt/vt) in a 56ml digestion vessel (Zarić et al., 2016). The digestion was conducted using a microwave digestion system (Anton Paar GmbH, Graz, Austria) based on the following scheme: 15min from room temperature to 200°C, 15min at 200°C, and cooling down to the room temperature (Zarić et al., 2016). The digestion solution was diluted to 10ml with deionized water in volumetric flasks and analyzed with an inductively coupled plasma optical emission spectrometer (PerkinElmer, Palo Alto, CA, United States).

### Honeybee Physiological Parameters

A live census was taken daily, by counting the number of mobile honeybees in each cage, to assess survivorship; any dead honeybees were removed after the census daily. The total mixture consumption of nano-La_2_O_3_ was calculated by subtracting the mass of the remaining mixture and water evaporation from the initial 4g mixture supplied daily. The 40% mass of total mixture consumption was divided by the number of surviving honeybees at each 24-h interval in each cage to calculate the pollen consumption per honeybee per day. Further, to determine the mass of the whole honeybee body, 12 live honeybees were randomly sampled from each cage and randomly separated into three groups of 4 honeybees, transferring into individual 50-ml sterile centrifuge tubes and weighed by the subtraction method with a digital balance (Mettler-Toledo, Shanghai, China; ± 0.001g).

### Gut Sample Collection

The entire exposure test lasted for 12days. After 6 and 12days, five live honeybees as individual replicates were sampled randomly from each cage and cold-anesthetized (−20°C, 1min). All immobilized honeybees were dissected on ice to collect the entire gut with flame-sterilized forceps under aseptic conditions. Each gut sample was placed into a 2-ml sterile centrifuge tube and immediately frozen in liquid nitrogen for subsequent DNA extraction and gut bacterial community analysis (Liu et al., 2019).

### DNA Extraction, PCR, and High-Throughput Sequencing

Gut DNA was extracted from the honeybee gut samples using the FastDNA SPIN Kit (MP Biomedicals, Santa Ana, CA, United States) according to manufacturer’s protocol. Extraction blanks, containing all the components except gut samples, were used as quality controls. The DNA from the whole gut of one bee was dissolved in 100μl TE buffer, quantified with NanoDrop 2000 (Thermo Scientific, Wilmington, DE, United States), and stored at −80°C until use.

The V3-V4 hypervariable regions of the bacterial 16S rRNA gene were amplified in triplicates with the primer set 338F (5′-ACTCCTACGGGAGGCAGCAG-3′) and 806R (5′-GGACTACHVGGGTWTCTAAT-3′). The oligonucleotides of six-base barcodes were incorporated with the forward and the reverse primers to distinguish sequencing samples. Polymerase Chain Reaction (PCR) was conducted in a 20μl reaction mixture, containing 4μl of 5×FastPfu Buffer, 2μl of 2.5mM dNTPs, 0.8μl of each primer (5μm), 0.4μl of FastPfu Polymerase (TransGen Biotech, Beijing, China), and 10ng of template DNA. Each reaction was performed under the following procedures: denaturation at 95°C for 3min, annealing for 25cycles (95°C for 30s, 55°C for 30s, and 72°C for 45s), and a final extension at 72°C for 10min. Reaction mixtures without DNA templates served as negative controls to test for contamination. The size and quality of PCR products were checked by TapeStation (Agilent Technologies, Santa Clara, CA, United States). The triplicate PCR products of each sample were pooled and purified using the AxyPrep DNA Gel Extraction Kit (Axygen Biosciences, Union City, CA, United States) and then quantified using the QuantiFluor-ST (Promega, Madison, WI, United States). After normalization in equimolar amounts, the purified amplicons were sequenced on an Illumina MiSeq platform (Illumina, San Diego, CA, United States) according to standard protocols.

### Bioinformatic Analysis

The raw sequencing data were processed using the Quantitative Insights Into Microbial Ecology. First, sequences were filtered according to Liu et al. (2020). Then, the high-quality sequences were merged using FLASH.^2^ Finally, the merged sequences were clustered into operational taxonomic units (OTUs) at 97% identity threshold using UPARSE,^3^ while chimeric sequences were removed using UCHIME. The OTUs were assigned to a taxonomic unit by RDP Classifier^4^ against the SILVA 16S rRNA database (Release 128)^5^ using a confidence threshold of 70%. The sequences were subsampled to the minimum depth (30,707) prior to analysis.

### Statistical Analysis

The “survival” of honeybees was estimated by the Kaplan-Meier (KM) method taking into account the numbers of survivors and dead honeybees, and the samples sacrificed during the study. The KM method is considered efficient and sufficiently general for estimating survival curve (Motta et al., 2018; Al Naggar et al., 2020). Statistical differences following various treatments were assessed by a log-rank paired test, and the *p* values were adjusted using a Bonferroni procedure. Cumulative pollen consumption indicated the total mass of pollen consumption from the first day to each time point, and the potential maximum honeybee pollen intake was estimated by fitting the cumulative pollen consumption to a first-order kinetic equation. Honeybee weight loss indicated the loss of body weight at each time point relative to the initial weight (Day 0), and the apparently zero-order rate of weight loss was obtained from a linear regression based on untransformed data. Comparisons of equation coefficients between the control and different treatments were achieved by bootstrapping (1000 times) followed by pairwise *t* test (Zhou et al., 2008).

One-way ANOVA with a *post hoc* least significant difference test was performed to test the differences among treatments. The contents of La were log-transformed for normality prior to ANOVA. The rarefied sample-OTU matrix was log-transformed to reduce the influence of highly abundant species (Anderson et al., 2006). Principal coordinates analysis (PCoA; using function “pcoa”), permutational multivariate analysis of variance (PERMANOVA; “adonis”), and the distance-based multivariate dispersion test (“betadisper”) were performed using VEGEN package in R,^6^ to assess the influence of nano-La_2_O_3_ on the gut bacterial community composition.

Due to the simple composition and significant inter-individual variation in the gut samples (Kwong and Moran, 2016), the genera that occurred in more than half of 55 samples were defined as the common ones. Spearman correlation was used to identify affected taxa whose relative abundance was significantly correlated (*p*<0.05) with nano-La_2_O_3_ exposure dose at day 6 and 12. Linear or exponential regression analysis was further conducted to examine the relationship between affected taxa and host physiological parameters (survival, cumulative pollen consumption, and weight loss) and nano-La_2_O_3_ exposure dose. Analyses were executed using R,^7^ SPSS (SPSS, Chicago, IL, United States), or SigmaPlot (Systat Software, Chicago, IL, United States).

## Results and Discussion

### La Content in Honeybees

**Figure 1:**
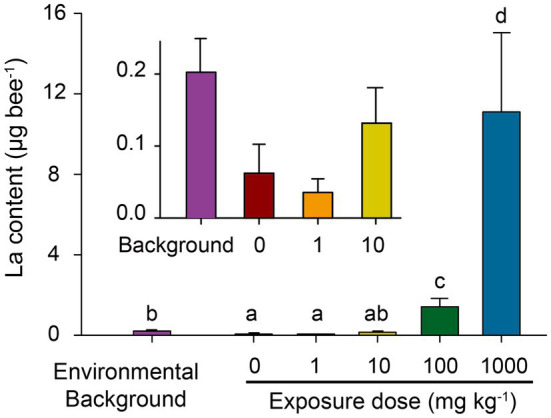
Environmental background of La content in wild honeybee (collected from field) and cumulative La residues in lab-reared honeybees after 12-day nano-La_2_O_3_ exposure. Error bars indicate the standard error of the mean (*n*=3). Different letters near bars indicate significant differences among samples (*p*<0.05; ANOVA-LSD; data were log-transformed for variance homogeneity prior to comparison).

Wild honeybees in the field were assayed to evaluate the environmental background of La in honeybees. Incubated honeybees with nano-La_2_O_3_ exposure were also assayed to assess treatment effects on uptake of dietary exposure of nano-La_2_O_3_. The background La residue (0.20μg bee^−1^) in wild honeybees was significantly higher than the lab-reared ones with no (exposure, 0mgkg^−1^; residual, 0.06μg bee^−1^) or low-dosage (1mgkg^−1^; 0.04μg bee^−1^) exposure of nano-La_2_O_3_ (*p*<0.05; Figure 1), suggesting that honeybees did suffer La exposure under field condition. For incubated honeybees, the La residues increased significantly with La_2_O_3_ exposure doses (Spearman’s *R*=0.86, *p*<0.001; Figure 1). Notably, La residue in the honeybees treated with medium dosage (10mgkg^−1^) nano-La_2_O_3_ exposure were comparable to the background La residue in wild honeybees (*p*>0.05; Figure 1). Therefore, the medium dosage of nano-La_2_O_3_ used in this study could be taken into account to predict the natural La exposure to honeybees, although it remains a challenge to characterize the complex forms of La during translocation in environmental matrices (Ma et al., 2011; Li et al., 2014). La residues in high (100mgkg^−1^) and the highest dosage (1,000mgkg^−1^) treatments were approximately 7 and 55 times that of wild honeybees (*p*<0.05; Figure 1). It is reported that high concentrations of La could result in irreversibly adverse impacts to plants and *Daphnia magna*, despite of its neutral effects on *Chlorella* sp. (Balusamy et al., 2015; Yue et al., 2017). In addition, the measured La residue was lower than the corresponding cumulative consumption (Supplementary Figure 1), indicating that nano-La_2_O_3_ was released to the intestinal environment and partially excreted through the gut. Therefore, we further conducted honeybee toxicity assays to explore the potential effects of nano-La_2_O_3_ on honeybee health and gut microbiota.

### Nano-La_2_O_3_ Deteriorates Honeybee Health

**Figure 2:**
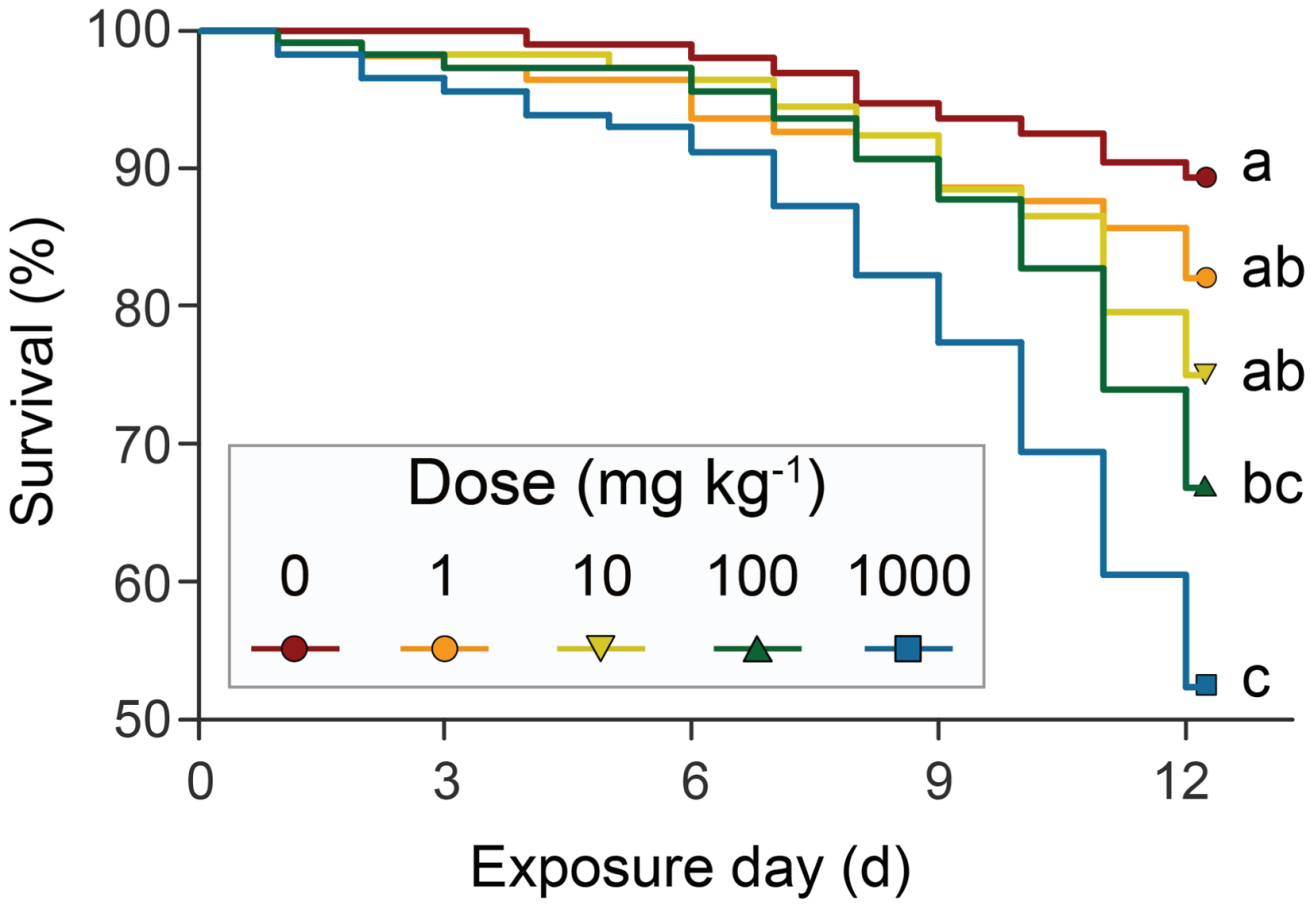
The Kaplan-Meier survival curves of honeybees under different concentrations of nano-La_2_O_3_ exposure. Survival was monitored and recorded each day for 12days. Different lower-case letters indicate significant differences among samples (log-rank (Mantel-Cox) paired test and Bonferroni correction, *p*<0.05). Detailed numbers of survivors are listed in Supplementary Table 1.

**Figure 3:**
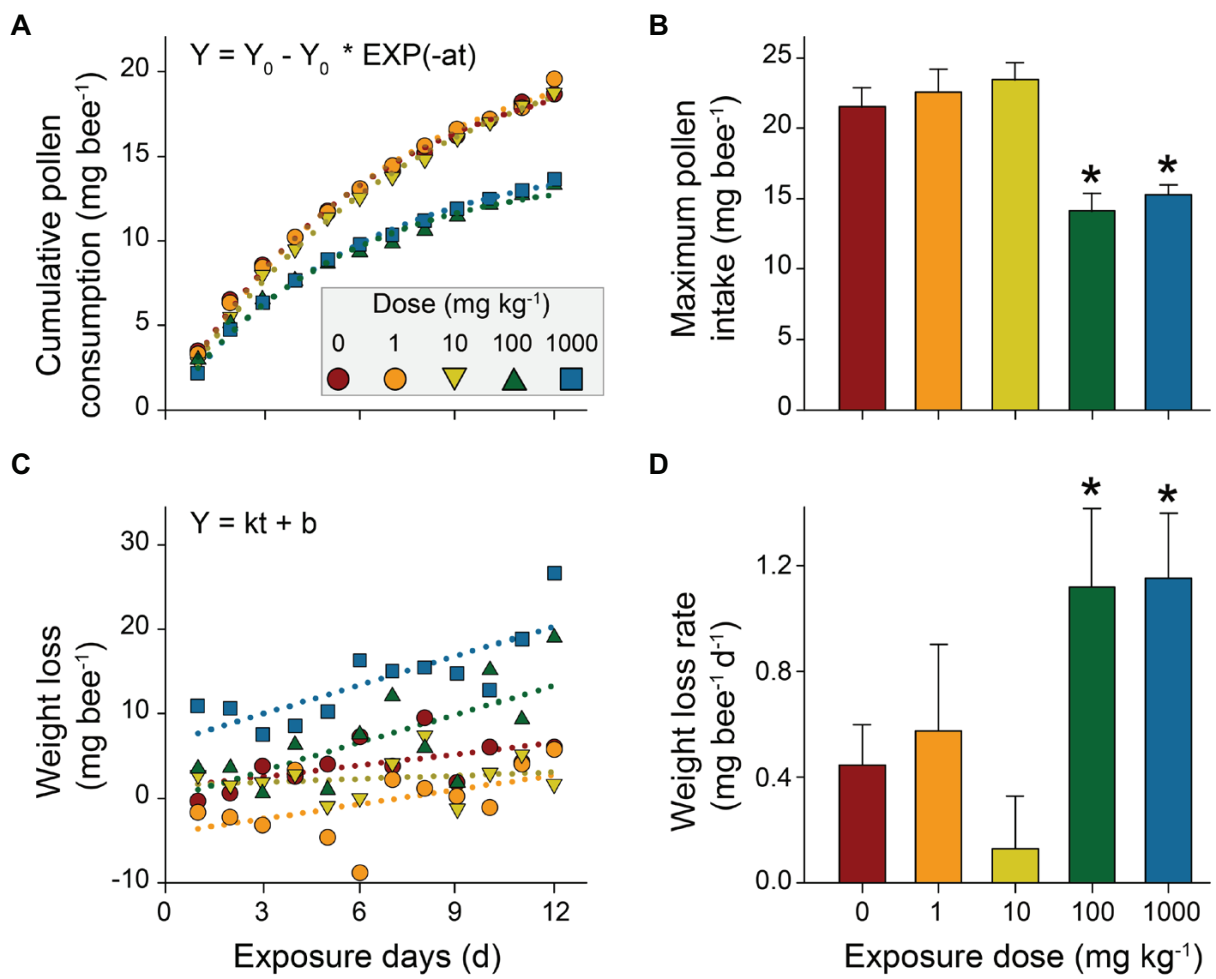
Effects of nano-La_2_O_3_ exposure on honeybees’ pollen consumption and weight loss. **(A)** cumulative pollen consumption per honeybee per day; **(B)** maximum pollen intake estimated by first-order kinetic fitting (Y_0_, mg bee^−1^, in A); **(C)** honeybee weight loss (the loss of the body weight per honeybee compared with the weight at day 0); and **(D)** weight loss rate estimated using linear regression (k, mg bee^−1^ d^−1^, in C). Error bars indicated 95% confidence intervals. ^*^indicates significant difference between treatment and control (*p*<0.05; tested using bootstrapping with 1000 randomizations). Detailed pollen consumption and body weight for each treatment are listed in Supplementary Tables 2 and 3.

The Kaplan-Meier survival curves of honeybees showed that survival significantly decreased in the high or highest NP concentration treatments compared to the control (*p*<0.001), while there were negligible decreases in low and medium treatments (*p*=1 and *p*=0.07, respectively; Figure 2; Supplementary Table 1). This suggests a dose-dependent toxic effect of nano-La_2_O_3_ on honeybee’s survival, which is in line with a previous study (Dabour et al., 2019). Previous studies indicated that nano-La_2_O_3_ is chronically toxic to the lung that can strip membrane phosphate groups in acidifying lysosomes and induce cellular and pulmonary damage (Li et al., 2014). Pollen provides most of the nutrients (e.g., proteins, amino acids, and lipids) for honeybee physiological development (Di Pasquale et al., 2013). The results revealed major differences across the time course of cumulative pollen consumption in different treatments (Figure 3A,B; Supplementary Table 2), indicating dose-dependent toxic effects of nano-La_2_O_3_ on honeybee nutrition intake (Glavan et al., 2017). In addition, there were positive relationships between weight loss and exposure days, such that the rate of the weight losses for the two highest dose treatments was significantly greater than that of the control (*p*<0.05, Figures 3C,D; Supplementary Table 3). Thus, exposure to sufficient doses of nano-La_2_O_3_ can decrease the cumulative body weight of honeybees. Body weight is a sensitive indicator of nutritional and energetic effects, which are tightly linked with gut microbiota (Zheng et al., 2017). Thus, the effect of nano-La_2_O_3_ exposure on the honeybee gut microbiota was worthy of investigation.

### Nano-La_2_O_3_ Causes Dysbiosis of Honeybee Gut Bacterial Communities

**Figure 4:**
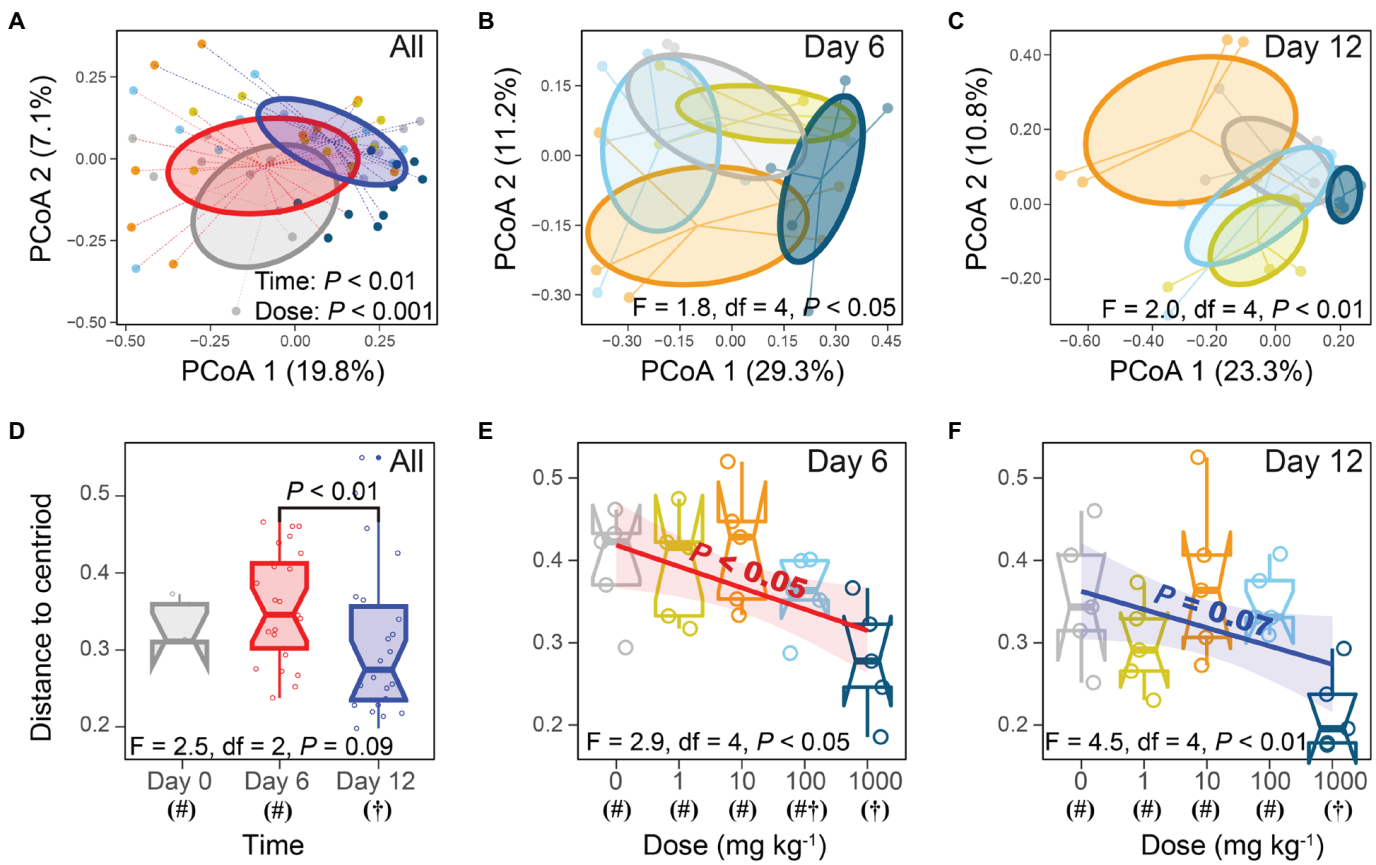
Nano-La_2_O_3_ exposure perturbs gut microbiota as evidenced by principal coordinates analysis **(A**–**C)**, nonparametric multivariate analysis of variance **(A**–**C)**, and multivariate dispersion test **(D**–**F)**. **(A,D)** analyses based on all samples; **(B,E)** samples of day 6; **(C,F)** samples of day 12. Different symbols (^#^ or ^†^) under x-axis in panel **(C**,**D)** indicate significant differences of gut bacterial community composition among treatments (*p*<0.05; multivariate dispersion test).

To access whether honeybee gut bacterial community will respond to nano-La_2_O_3_ exposure, we extracted gut DNA (Supplementary Figure 2) and conducted bacterial 16S rRNA gene sequencing. The results of PCoA, PERMANOVA, and distance-based multivariate dispersion showed that nano-La_2_O_3_ exposure caused significant shift of gut bacterial community composition (*p* <0.05; Figures 4A–F), with or without the consideration of the effect of exposure days (day 6 and 12; *p* <0.05; Figures 4A,D). These results suggest that Nano-La_2_O_3_ exposure can cause significant honeybee gut bacterial compositional dysbiosis in a relative short term of within 6-day exposure. When the dose of nano-La_2_O_3_ exposure was assessed, a gradual shift of gut bacterial community composition with increasing exposure dose of nano-La_2_O_3_ was observed at both day 6 (linear regression, *p* <0.05) and day 12 (*p* =0.07), while the significant effects were only observed for the highest dose (1,000mgkg^−1^) at both days 6 and 12 as evidenced by the pairwise comparison of gut community compositional differences (*p* <0.05; multivariate dispersion test; Figures 4E,F). These further suggest the dose-dependent effect of nano-La_2_O_3_ on honeybee gut microbiota and that a relative high threshold of nano-La_2_O_3_ exposure (e g., a diet concentration of 100–1,000mgkg^−1^ or an intake rate of 0.1–1.0μg bee^−1^ day^−1^; Figure 1) are necessary to generate substantial and sharp shifts of gut microbial communities in a short term (within 6days).

**Figure 5:**
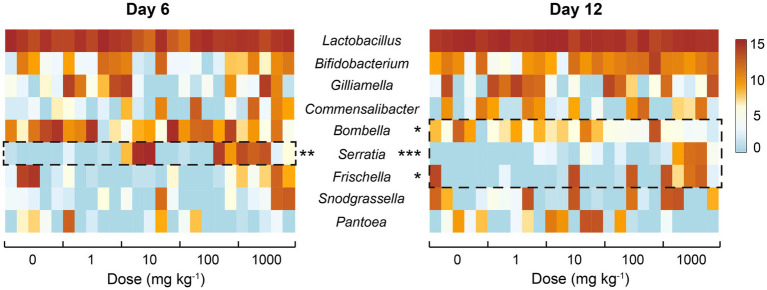
Heatmap illustrating the relative sequence abundances (log2-transformed; 30,707 depth basis) of common bacterial genera in the guts of honeybees of different treatments. Asterisk indicate significant relationship (Spearman’s correlation) between the relative abundances of core genera and exposure dose of nano-La_2_O_3_ (^*^p<0.05, ^**^p<0.01, ^***^p<0.001).

Besides the overall shift of honeybee gut bacterial communities, the response of specific members within the communities to nano-La_2_O_3_ exposure was also examined. In total, 64 genera comprised of 96 OTUs were identified, while the gut bacterial community composition was mainly dominated by nine genera (Figure 5), accounting for *ca*. 95.87% total abundances of the whole communities. Notably, the genera *Serratia*, *Frischella*, and *Bombella* were significantly related with nano-La_2_O_3_ exposure dose (*p*<0.05; Figure 5). Specifically, nano-La_2_O_3_ exposure enriched gut pathogen genera *Serratia* and *Frischella*, among which, the genus *Serratia* was found to be the most sensitive taxa whose relative abundance increased most significantly with increasing exposure dose (*p*<0.001 at day 12, Figure 5). It is important to note that the genus *Serratia* is an intrinsically opportunistic pathogen which becomes highly abundant when hosts are stressed or become diseased (Glavan et al., 2017; Raymann et al., 2018). The genus *Serratia* contains antimicrobial resistance genes acquired through horizontal gene transfer, which likely contributes to its high tolerance against stresses (Sandner-Miranda et al., 2018). Hence, the opportunistic pathogen *Serratia* has a survival advantage relative to other bacteria, for example, when nano-La_2_O_3_ induces cellular phospholipid damage and bacterial death (Li et al., 2014). In addition, the genus *Frischella* is often reported as a rare gut bacterial taxa that is less abundant and irregularly occurring (Kwong and Moran, 2016). The abundance of *Frischella* in our study varied largely across individual honeybees, but positively correlated with the exposure dose of nano-La_2_O_3_ at day 12 (Figure 5). This is supported by the fact that the *Frischella* is an opportunistic pathogen that can establish in a specific niche, for example, when the host suffers tissue damage and pathogen invasion (Maes et al., 2016; Emery et al., 2017). We also found that the typically acetic acid forming genus *Bombella* (Yun et al., 2017) was negatively related to nano-La_2_O_3_ exposure at day 12 (Figure 5).

There are specific relationships between environmental stressors, honeybee gut microbiota, and honeybee colonial resistance to pathogens (Doublet et al., 2015; Bonilla-Rosso and Engel, 2018; Radziwill-Bienkowska et al., 2018). Nano-La_2_O_3_ could dissolve in the gut and strip phosphates from the phospholipids on bacterial membranes (Zheng et al., 2019), which could cause cell damage and impose enhanced selection pressure on gut bacteria. When the bacterial community is unstable, honeybee colony disease resistance may decrease with the thrive of existing gut pathogens (Maes et al., 2016). The significance here is that the enteric pathogens, which were involved in specific and functionally distinct interactions within the bacterial community, could cause gut dysbiosis. The prevalence of pathogenic *Serratia* and *Frischella* indicate a disrupted gut homeostasis, thus serving as a diagnostic signature of dysbiosis (Emery et al., 2017; Raymann et al., 2018). Therefore, nano-La_2_O_3_ exposure may not only induce dysbiosis of honeybee gut bacterial communities, but also enhance the competitive advantages of pathogens.

### Nano-La2O3 Exposure Affects Honeybee Health by Causing Dysbiosis of Its Gut Bacterial Communities

**Figure 6:**
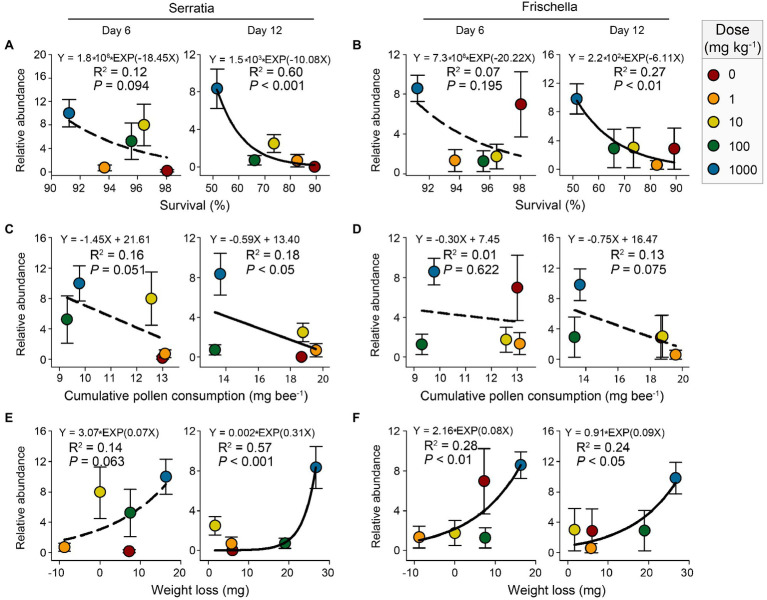
Linear or exponential regressions showing the relationships between honeybee physiological parameters (survival, cumulative pollen consumption, and weight loss) and the relative abundances (log2-transformed) of genera *Serratia* **(A**,**C**,**E)** and *Frischella* **(B**,**D**,**F)** after 6-day and 12-day nano-La_2_O_3_ exposures. Error bars indicate the standard error of the mean (*n*=5). The solid and dotted lines represent significant (*p*<0.05) and nonsignificant (*p*>0.05) relationships, respectively.

Gut microbiota play crucial roles in host health (Engel et al., 2016; Zhang et al., 2020). The relationships between the abundances of main bacterial taxa and the physiological parameters of honeybees (survival, cumulative pollen consumption, and weight loss) were assessed with linear or exponential regression (Supplementary Table 4). The result showed that the abundances of pathogenic *Serratia* and *Frischella* were significantly related to honeybee physiological parameters at either day 6 or day 12 (*p*<0.05; Figure 6).

**GRAPHICAL ABSTRACT:**
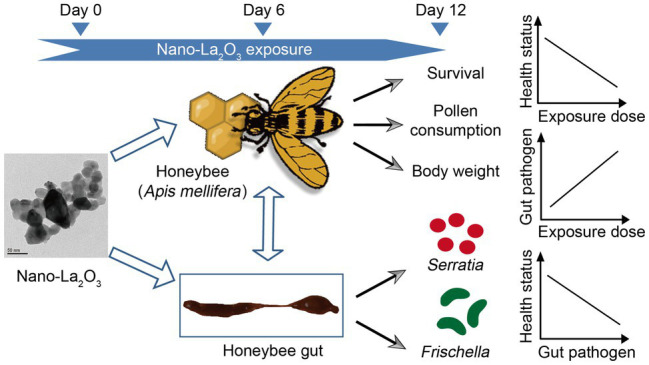
The effects of nano-La_2_O_3_ on honeybee physiology and gut bacterial communities are additive: nano-La_2_O_3_ can deteriorate honeybee health potentially by enriching for pathogens in gut bacterial communities.

Our result showed a significant decrease of honeybee survival with the increasing abundances of the pathogenic genera *Serratia* and *Frischella* at day 12 (Figures 6A,B). This is in line with previous studies, showing that the enrichment of pathogens, for example, the genera *Serratia* and *Frischella*, deteriorates host development and increases host mortality (Doublet et al., 2015;Maes et al., 2016; Raymann et al., 2018). Our results, as well as the previous evidence, imply that the response of the gut microbiota and the specific functional taxa, including the pathogenic ones, may act important roles in mediating the effects of contaminants, such as nano-La_2_O_3_ assessed in this study, on host health (Raymann et al., 2017; Motta et al., 2018). Notably, these correlations did not provide conclusive evidence that the enrichment of the pathogenic *Serratia* and *Frischella* directly cause honeybee death, but they are suggestive. Further, perhaps *in vivo*, infection experiments are needed to establish the virulence of pathogens from various contaminant exposures and to assess whether pathogenesis derives directly from contaminant-promoted thrive of pathogen or indirectly from contaminant-induced inhibition of beneficial taxa.

Gut dysbiosis induces dramatic effects on honeybee health (Hamdi et al., 2011). Gut microbiota affect host weight by mediating host nutritional physiology (e.g., vitellogenin level; Zheng et al., 2017). Hence, gut dysbiosis can cause metabolic disorders and impair host development through altering hormone production (Cryan and Dinan, 2012). In this study, the abundance of *Serratia* was significantly related to cumulative pollen consumption and weight loss at day 12 (Figures 6C,E). The abundance of *Frischella* exponentially increased, while the body weight decreased under nano-La_2_O_3_ exposure (Figures 6D,F). According to these results, an inference is that the markers of gut dysbiosis, pathogenic *Serratia* and *Frischella*, may affect hormonal signaling-related gene expression and subsequently induce pathophysiological responses (e.g., impaired nutrition intake and body weight; Maes et al., 2016; Raymann et al., 2018). However, testing this hypothesis requires studying the relationship between hormone gene expression and gut pathogen abundance. Generally, it may be difficult to disentangle the effects of nano-La_2_O_3_ on gut bacterial communities independent of the direct effects on the host, which may, in turn, alter the tolerance to pathogens (Raymann et al., 2017). For example, nano-La_2_O_3_ exposure inhibited honeybee pollen intake and thus affected the resistance threshold of honeybees to pathogen stress (Di Pasquale et al., 2013). Although several studies have explored how contaminant-induced shifts in gut microbiota affect host physiology (Raymann et al., 2017; Motta et al., 2018), evidence of direct effects is currently lacking. In the future, it would be important to investigate how changes in honeybee health following environment disturbances may trigger changes to gut microbiota.

## Conclusion

In summary, this study examined the effects of nano-La_2_O_3_ on honeybee health and gut bacterial communities. Our results provide evidence that nano-La_2_O_3_ exerted dose-dependent detrimental effects on honeybee physiology as reflected by the decrease in honeybee survival, pollen consumption, and body weight. Further, the exposures of 0 to 100mgkg^−1^ nano-La_2_O_3_ had no significant effects on gut bacterial community, while the exposure dose of 1,000mgkg^−1^ caused a significant community compositional shift. Besides, the specific genera within the community, including the pathogenic *Serratia* and *Frischella*, and the digestion-related bacteria *Bombella*, also responded significantly to nano-La_2_O_3_ exposure. Moreover, honeybee physiological impairments were significantly related to the enrichment of *Serratia* and *Frischella*. Collectively, these findings suggest that pathogen enrichment and gut dysbiosis may be at least partially responsible for adverse effects of nano-La_2_O_3_ exposure to honeybee health, thus extending our knowledge regarding the effects of nano-La_2_O_3_ on honeybee (*Apis mellifera*).

## Data Availability Statement

The 16S rRNA datasets generated in this study can be found in the SRA archive in GenBank under the BioProject PRJNA764692.

## Ethics Statement

This study was reviewed and approved by the ethics committee of Institute of Apicultural Research, Chinese Academy of Agricultural Sciences (IAR, CAAS).

## Author Contributions

YG and Y-JL proposed the project and designed the experiments. Q-YD, ZJ, Y-YW, and Y-JL performed the exposure experiment and collected honeybee physiological data. X-TB, JW, and Y-JL collected the sequencing data. TX characterized the nanoparticles. X-TB and ZJ analyzed and visualized the data. X-TB and YG wrote the original draft. QZ, TX, BX, ZJ, and PH reviewed and edited the manuscript. All authors contributed to the data interpretation and paper writing.

## Funding

This work was supported by National Natural Science Foundation of China (41671254 and 31772683), Chinese Academy of Agricultural Sciences (Elite Youth Program to Y-JL), and State Key Laboratory of Urban and Regional Ecology (SKLURE2017-1\137).

## Conflict of Interest

The authors declare that the research was conducted in the absence of any commercial or financial relationships that could be construed as a potential conflict of interest.

## Publisher’s Note

All claims expressed in this article are solely those of the authors and do not necessarily represent those of their affiliated organizations, or those of the publisher, the editors and the reviewers. Any product that may be evaluated in this article, or claim that may be made by its manufacturer, is not guaranteed or endorsed by the publisher.

## Footnotes

^1^ http://iar.caas.cn/en/

^2^ http://ccb.jhu.edu/software/FLASH/

^3^ http://drive5.com/uparse/

^4^ http://rdp.cme.msu.edu/

^5^ http://www.arb-silva.de/

^6^ https://www.r-project.org/

^7^ http://www.r-project.org/
