# Supplementary material for: Nano-La2O3 Induces Honeybee (Apis mellifera) Death and Enriches for Pathogens in Honeybee Gut Bacterial Communities
Source: Front Microbiol. 2021 Dec 2;12:780943. doi: 10.3389/fmicb.2021.780943 (PMC8674717; doi:10.3389/fmicb.2021.780943)
Supplement: Supplementary file 1 [file Data_Sheet_1.docx]

Supplementary information for

Nano-La_2_O_3_ induces honeybee (Apis mellifera) death and enriches for pathogens in honeybee gut bacterial communities

Yong-Jun Liu^1^†*, Zhongwang Jing^23^†, Xue-Ting Bai^23^†, Qing-Yun Diao^1^, Jichen Wang^23^, Yan-Yan Wu^1^, Qing Zhao^4^, Tian Xia^5^, Baoshan Xing^6^, Patricia A. Holden^7^, Yuan Ge^ab^*

^1^ Key Laboratory of Pollinating Insect Biology, Institute of Apicultural Research, Chinese Academy of Agricultural Sciences, Beijing 100093, China

^2^ State Key Laboratory of Urban and Regional Ecology, Research Center for Eco-Environmental Sciences, Chinese Academy of Sciences, Beijing 100085, China

^3^ University of Chinese Academy of Sciences, Beijing 100049, China

^4^ Key Laboratory of Pollution Ecology and Environmental Engineering, Institute of Applied Ecology, Chinese Academy of Sciences, Shenyang 110016, China

^5^ Division of NanoMedicine, Department of Medicine, University of California, Los Angeles, California 90095, United States

^6^ Stockbridge School of Agriculture, University of Massachusetts, Amherst, Massachusetts 01003, United States

^7^ Bren School of Environmental Science & Management, and Earth Research Institute, University of California, Santa Barbara, California 93106, United States

† These authors have contributed equally to this work and share first authorship

*** Correspondence:**

Yuan Ge, Email: [yuange@rcees.ac.cn](mailto:yuange@rcees.ac.cn)

Yong-Jun Liu: [liuyongjun@caas.cn](mailto:liuyongjun@caas.cn)

This file contains Figs. S1-2 and Tables S1-5.


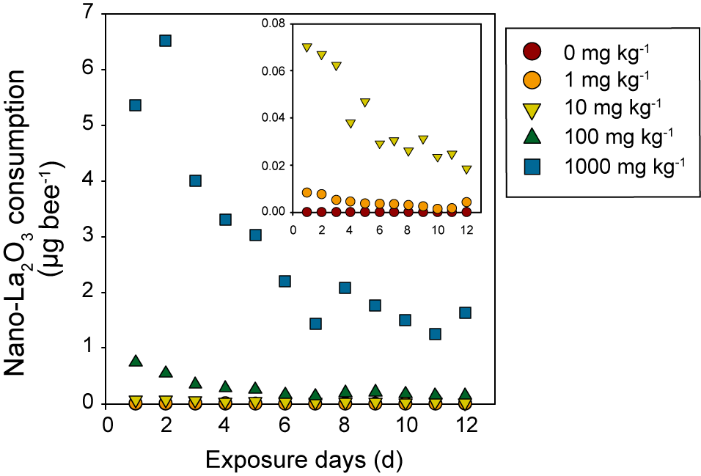


**Fig. S1.** Nano-La_2_O_3_ consumption each day.


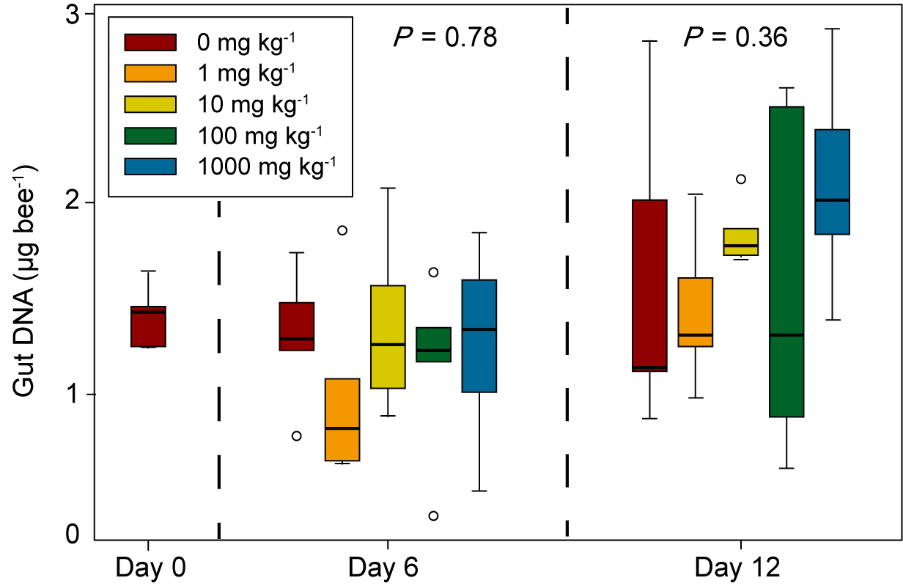


**Fig. S2.** Total gut DNA contents of honeybee samples at day 0, 6 and 12. The exposure doses were 0 (control), 1 (low), 10 (medium), 100 (high) or 1000 (highest) mg kg^-1^ of nano-La_2_O_3_. The box plot denotes median (horizontal line within the box), 25th (bottom edge of the box), 75th (top edge of the box), the highest datum (error bars above the box), lowest datum (error bars below the box).

**Table S1.** The numbers of survivors and dead honeybees each day during the pre-incubation and nano-La_2_O_3_ exposure for the control and each treatment.

|  | Nano-La_2_O_3_ exposure dose (mg kg^-1^) | Pre-incubation (7 days) | | | | | | |  | Nano-La_2_O_3_ exposure (12 days) | | | | | | | | | | | |  |
| --- | --- | --- | --- | --- | --- | --- | --- | --- | --- | --- | --- | --- | --- | --- | --- | --- | --- | --- | --- | --- | --- | --- |
|  |  |  |  |  |  |  |  |  |  | Day 0 | Day 1 | Day 2 | Day 3 | Day 4 | Day 5 | Day 6 | Day 7 | Day 8 | Day 9 | Day 10 | Day 11 | Day 12 |
| Survivor | 0 | 120 | 120 | 120 | 119 | 115 | 115 | 115 |  | 102 | 102 | 102 | 102 | 101 | 101 | 90 | 89 | 87 | 86 | 85 | 83 | 71 |
|  | 1 | 120 | 120 | 120 | 118 | 115 | 115 | 112 |  | 111 | 110 | 109 | 108 | 107 | 107 | 93 | 92 | 90 | 88 | 87 | 85 | 70 |
|  | 10 | 120 | 120 | 120 | 118 | 115 | 115 | 113 |  | 112 | 111 | 110 | 110 | 110 | 109 | 97 | 95 | 93 | 89 | 87 | 80 | 63 |
|  | 100 | 120 | 120 | 120 | 120 | 118 | 116 | 116 |  | 113 | 112 | 111 | 110 | 110 | 110 | 97 | 95 | 92 | 89 | 84 | 75 | 56 |
|  | 1000 | 120 | 120 | 120 | 119 | 115 | 115 | 115 |  | 114 | 112 | 110 | 109 | 107 | 106 | 92 | 88 | 83 | 78 | 70 | 61 | 41 |
| Dead | 0 |  | 0 | 0 | 0 | 4 | 0 | 0 |  | 2 | 0 | 0 | 0 | 1 | 0 | 1 | 1 | 2 | 1 | 1 | 2 | 1 |
|  | 1 |  | 0 | 0 | 2 | 3 | 0 | 3 |  | 1 | 1 | 1 | 1 | 1 | 0 | 3 | 1 | 2 | 2 | 1 | 2 | 3 |
|  | 10 |  | 0 | 0 | 2 | 3 | 0 | 2 |  | 1 | 1 | 1 | 0 | 0 | 1 | 1 | 2 | 2 | 4 | 2 | 7 | 6 |
|  | 100 |  | 0 | 0 | 0 | 2 | 2 | 0 |  | 3 | 1 | 1 | 1 | 0 | 0 | 2 | 2 | 3 | 3 | 5 | 9 | 8 |
|  | 1000 |  | 0 | 0 | 1 | 4 | 0 | 0 |  | 1 | 2 | 2 | 1 | 2 | 1 | 2 | 4 | 5 | 5 | 8 | 9 | 9 |

**Table S2.** Pollen consumption per honeybee each day.

|  | Nano-La_2_O_3_ exposure dose (mg kg^-1^) | Nano-La_2_O_3_ exposure (12 days) | | | | | | | | | | | |
| --- | --- | --- | --- | --- | --- | --- | --- | --- | --- | --- | --- | --- | --- |
|  |  | Day  1 | Day  2 | Day  3 | Day  4 | Day  5 | Day  6 | Day  7 | Day  8 | Day  9 | Day  10 | Day  11 | Day  12 |
| Pollen consumption (mg bee^-1^ day^-1^) | 0 | 3.486 | 3.029 | 2.022 | 1.651 | 1.559 | 1.242 | 1.109 | 1.074 | 1.038 | 0.975 | 1.003 | 0.483 |
|  | 1 | 3.302 | 3.028 | 2.081 | 1.785 | 1.480 | 1.409 | 1.351 | 1.184 | 0.984 | 0.581 | 0.676 | 1.711 |
|  | 10 | 2.816 | 2.677 | 2.499 | 1.520 | 1.880 | 1.169 | 1.218 | 1.049 | 1.255 | 0.941 | 0.992 | 0.738 |
|  | 100 | 2.968 | 2.166 | 1.378 | 1.123 | 1.026 | 0.639 | 0.546 | 0.761 | 0.834 | 0.704 | 0.575 | 0.595 |
|  | 1000 | 2.147 | 2.610 | 1.602 | 1.322 | 1.212 | 0.879 | 0.576 | 0.835 | 0.706 | 0.603 | 0.499 | 0.655 |

**Table S3.** Whole body mass per honeybee each day.

|  | Nano-La_2_O_3_ exposure dose (mg kg^-1^) | Nano-La_2_O_3_ exposure (12 days) | | | | | | |
| --- | --- | --- | --- | --- | --- | --- | --- | --- |
|  |  | Day  0 | Day  1 | Day  2 | Day  3 | Day  4 | Day  5 | Day  6 |
| Weight  (g bee^-1^) | 0 | 0.111±0.004 | 0.111±0.002 | 0.110±0.003 | 0.107±0.003 | 0.108±0.007 | 0.107±0.000 | 0.104±0.004 |
|  | 1 | 0.108±0.005 | 0.110±0.004 | 0.110±0.013 | 0.111±0.003 | 0.105±0.001 | 0.113±0.006 | 0.117±0.006 |
|  | 10 | 0.108±0.004 | 0.106±0.005 | 0.107±0.004 | 0.106±0.003 | 0.106±0.003 | 0.109±0.004 | 0.108±0.004 |
|  | 100 | 0.111±0.008 | 0.107±0.001 | 0.107±0.007 | 0.110±0.005 | 0.105±0.006 | 0.110±0.005 | 0.103±0.004 |
|  | 1000 | 0.117±0.007 | 0.106±0.002 | 0.106±0.002 | 0.109±0.005 | 0.108±0.004 | 0.107±0.002 | 0.101±0.002 |
|  | Nano-La_2_O_3_ exposure dose (mg kg^-1^) | Nano-La_2_O_3_ exposure (12 days) | | | | | | |
|  |  |  | Day  7 | Day  8 | Day  9 | Day  10 | Day  11 | Day  12 |
| Weight  (g bee^-1^) | 0 |  | 0.107±0.002 | 0.101±0.002 | 0.109±0.004 | 0.105±0.000 | 0.107±0.000 | 0.105±0.002 |
|  | 1 |  | 0.106±0.003 | 0.107±0.004 | 0.108±0.005 | 0.109±0.003 | 0.104±0.001 | 0.102±0.004 |
|  | 10 |  | 0.104±0.002 | 0.101±0.007 | 0.110±0.004 | 0.105±0.004 | 0.103±0.001 | 0.107±0.002 |
|  | 100 |  | 0.099±0.002 | 0.105±0.001 | 0.109±0.004 | 0.096±0.007 | 0.102±0.002 | 0.092±0.004 |
|  | 1000 |  | 0.102±0.002 | 0.101±0.002 | 0.102±0.000 | 0.104±0.002 | 0.098±0.001 | 0.090±0.007 |

**Table S4.** Linear or exponential regression analysis showing the relationships between the host physiological parameters (survival, cumulative pollen consumption and weight loss) and the abundances of affected taxa (Serratia, Frischella, and Bombella).

|  |  |  |  | Regression analysis | |
| --- | --- | --- | --- | --- | --- |
|  |  |  |  | *R^2^* value | *P* value |
| Day6 | *Serratia* | vs. | Survival | 0.117 | 0.094 |
|  | *Frischella* |  |  | 0.072 | 0.195 |
|  | *Bombella* |  |  | 0.099 | 0.127 |
|  | *Serratia* | vs. | Cumulative pollen consumption | 0.156 | 0.051 |
|  | *Frischella* |  |  | 0.011 | 0.622 |
|  | *Bombella* |  |  | 0.001 | 0.870 |
|  | *Serratia* | vs. | Weight loss | 0.142 | 0.063 |
|  | *Frischella* |  |  | 0.283 | **0.006**** |
|  | *Bombella* |  |  | 0.002 | 0.825 |
| Day12 | *Serratia* | vs. | Survival | 0.604 | **0.000***** |
|  | *Frischella* |  |  | 0.268 | **0.008**** |
|  | *Bombella* |  |  | 0.106 | 0.113 |
|  | *Serratia* | vs. | Cumulative pollen consumption | 0.184 | **0.032*** |
|  | *Frischella* |  |  | 0.132 | 0.075 |
|  | *Bombella* |  |  | 0.090 | 0.146 |
|  | *Serratia* | vs. | Weight loss | 0.569 | **0.000***** |
|  | *Frischella* |  |  | 0.241 | **0.013*** |
|  | *Bombella* |  |  | 0.149 | 0.057 |
| *, **, and *** indicate *P* ＜ 0.05, 0.01 and 0.001, respectively. | | | | | |
